# Sexual differences in food preferences in the white stork: an experimental study

**DOI:** 10.1007/s00114-017-1457-5

**Published:** 2017-04-07

**Authors:** Zbigniew Kwieciński, Zuzanna M. Rosin, Łukasz Dylewski, Piotr Skórka

**Affiliations:** 1 Department of Avian Biology and Ecology, Faculty of Biology, Adam Mickiewicz University, Umultowska 89, 61-614 Poznań, Poland; 2 Department of Cell Biology, Adam Mickiewicz University, Umultowska 89, 61-614 Poznań, Poland; 3 Institute of Zoology, Poznań University of Life Sciences, Wojska Polskiego 71C, 60-625 Poznań, Poland; 4 Institute of Nature Conservation, Polish Academy of Sciences, Mickiewicza 33, 31-120 Kraków, Poland

**Keywords:** Cafeteria test, Diet, Food preferences, Sex differences

## Abstract

Sex differences in the foraging ecology of monomorphic species are poorly understood, due to problems with gender identification in field studies. In the current study, we used experimental conditions to investigate the food preferences of the white stork *Ciconia ciconia*, an opportunistic species in terms of food, but characterised by a low level of sexual dimorphism. During a 10-day experiment, 29 individuals (20 females and 9 males) were studied by means of a ‘cafeteria test’ in which the storks’ diet consisted of mammals, birds, fish, amphibians, insects and earthworms. The storks preferred food characterised by high calorific and protein values such as mammals, birds and fish. Sexes differed strongly in their preferences; males preferred mammals, whereas females preferred birds. Moreover, females consumed insects and earthworms less often than males. Interestingly, males spent significantly less time foraging than females. We have demonstrated that the white stork exhibits clear sexual differences in food preferences which are mostly attributable to differences in parental duties, physiology and anatomy.

**Electronic supplementary material:**

The online version of this article (doi:10.1007/s00114-017-1457-5) contains supplementary material, which is available to authorized users.

## Introduction

Food acquisition is one of the major factors determining individual fitness and survival in animal populations (Kendeigh et al. 1977; Walsberg 1983). Traditionally, it is believed that species characterised by a high degree of sexual dimorphism differ substantially between sexes in diet composition (Barton and Houston 1993; Hailey et al. 1998; Hilton et al. 1999; Slagsvold et al. 2010). These differences may result from competitive avoidance, differences in physiology and sex-specific nutrient requirements due to differences in parental effort (Hawkins 1986; Halupka 1994; Durant et al. 2000; Deeming 2002b; Neger 2006; Durant et al. 2010). Sexual dimorphism may be also related to food specialisation, with a higher degree of the latter in more dimorphic species (Tortosa and Redondo 1992; Temeles et al. 2000). However, there is also growing evidence in species with little or no sexual dimorphism that males and females may differ in several aspects of foraging ecology such as diet composition, selection of foraging areas and, finally, parental feeding (Stephens and Krebs 1986; Morrison et al. 1990; Dziewiaty 1992; Deeming 2002a; Stephens et al. 2007; Janiszewski et al. 2014). Thus, a study on a monomorphic species may shed light on the mechanisms leading to food-niche differences between sexes.

One such monomorphic species is the white stork *Ciconia ciconia*, an opportunistic feeder. However, the question of whether this species is characterised by sex-related food preferences has not been tested to date (Latus and Kujawa 2005; Djerdali et al. 2008). Numerous authors researching the diet of the white stork or of closely related species such as the wood stork *Mycteria americana* have pointed out that these diets are dependent on climatic conditions, habitat type, prey densities and availability, suggesting a high degree of plasticity in food choice rather than a strong preference for any particular food type (Krapivny 1957; Pinowski et al. 1991; Gonzalez 1997; Antczak et al. 2002; Tryjanowski and Kuźniak 2002; Tryjanowski et al. 2005; Tryjanowski and Hromada 2005; Zduniak 2005; Profus 2006; Kosicki et al. 2006; Ciach and Kruszyk 2010; Chenchouni et al. 2015; Chenchouni 2016; Orłowski et al. 2016). These studies were done in field conditions, which preclude unambiguous inferences about food selectivity. Only studies under controlled conditions in which the availability and nutritional quality of food is known and environmental conditions are uniform enable differentiation between real food preferences and those observed under natural conditions, which are obscured by other factors.

Despite minor morphological differences between white stork males and females, there are at least two arguments for expecting sexual differences in diet and foraging patterns. First, recent studies have shown that although dimorphism in body size is slight (males are about 12.5% heavier than females), the sexes differ significantly in intestinal length and digestive performance (Kwieciński and Tryjanowski 2009). Secondly, white stork females and males differ strongly in parental duties, with incubation performed mainly by females and general duties carried out by males (Bocheński and Jerzak 2006; Wuczyński 2012).

Therefore, the aim of this study was to answer the following questions: (1) Does the white stork show any food preferences (measured by preference index, sequence of choices and duration of foraging)? (2) What types of food do white storks prefer? (3) Are there any sex differences in these preferences?

## Materials and methods

### Study animals and study design

The study was conducted at the Poznań Zoological Garden between 2004 and 2005 (in May and June of each year). We investigated 29 wild-born white stork individuals (5 males and 7 females in 2004 and 4 males and 13 females in 2005) acquired by the zoo due to various accidents, mostly damages of the wings (Kwieciński et al. 2006a, b). After a 2-week medical curing, birds were taken to the common enclosure (300 m^2^) where they could freely move and get familiar with laboratory conditions. They adapted to the enclosure quickly because they started foraging by themselves within 2 days. Birds spent 8 months in captivity before the experiments; thus, they were fully familiar with the enclosure.

**Fig. 1:**
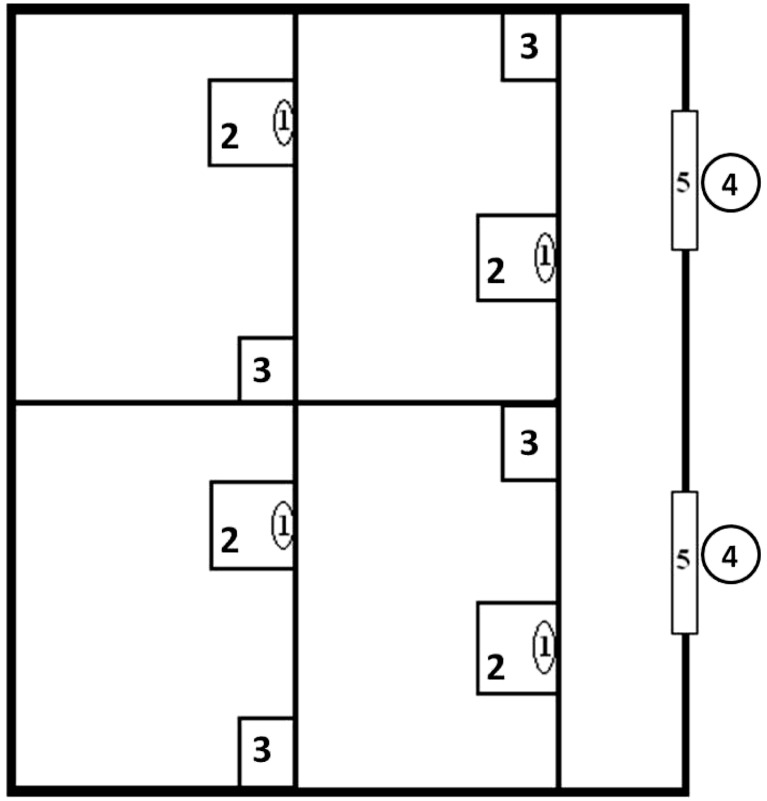
A sketch of the boxes used for the research on food preferences. *1*—mirror, *2*—plastic trays, *3*—water, *4*—observer location, *5*—window

All individuals were adult (age was determined on the basis of beak and leg colour). The mean (±SD) mass of males and females was 3230 (±360) and 2797 (±340) g, respectively. Their sex was determined using DNA techniques (Ćwiertnia et al. 2006). The sex of tested birds was not known to observers in the zoo until the feeding experiment was finished; hence, our results were not affected by prior knowledge of the birds’ sex during the experiment. During the experiments, birds were kept inside individual boxes with areas of ca. 10 m^2^ (Fig. 1). The boxes were enclosed with wire nets to enable easy observation from a distance. Each bird was individually marked with coloured rings, and a corresponding number was placed at a visible location in the box to prevent mistakes in recording (for more information on cage structure and observation distances, see Kwieciński et al. 2006a, b).

### Experimental procedure

A single experiment lasted 10 days for each bird. To reduce the stress caused by separation as well as by close contact with humans, four to five birds took part in the experiment simultaneously (Fig. 1). Each individual was tested only once during the research. The birds were offered a varied diet consisting of six types of prey:

- Mammals: the house mouse *Mus musculus* (captive-bred), the bank vole *Myodes glareolus*, the common vole *Microtus arvalis*, the striped field mouse *Apodemus agrarius*, the yellow-necked mouse *Apodemus flavicollis* and the wood mouse *Apodemus sylvaticus*
- Birds: 1-day-old red junglefowl chicks *Gallus gallus*, young grey partridge chicks *Perdix perdix* and ring-necked pheasant chicks *Phasianus colchicus*
- Amphibians: the common frog *Rana temporaria* and the moor frog *Rana arvalis*
- Fish: the sprat *Sprattus sprattus*, the European perch *Perca fluviatilis*, the common roach *Rutilus rutilus* and the crucian carp *Carassius carassius*
- Insects: the crickets *Acheta domesticus* and *Gryllus bimaculatus* and coleopterans (family Carabidae: *Carabus nemoralis*, *Carabus granulatus* as well as smaller beetles from the families Silphidae, Neerophoridae and Tenebrionidae)
- Annelids (earthworms *Lumbricus* spp*.*)

The diet items offered to birds were chosen based on published data on the diet of the white stork in the wild (Cramp and Simmons 1988; Pinowska and Pinowski 1989; Pinowska et al. 1991; Pinowski et al. 1991; Mužinić and Rašajski 1992; Antczak et al. 2002; Kosicki et al. 2006; Chenchouni et al. 2015; Orłowski et al. 2016). The food was presented in shallow plastic containers, according to the procedures associated with a ‘cafeteria test’, at the same time every day, i.e. at about 4 p.m. (Rychlik and Jancewicz 2002; Bergvall and Leimar 2005). Each examined individual was offered 200 g of each type of prey (mammals, birds, etc.). Water was available ad libitum and changed daily. Food items were counted and weighed separately for each food type using a Pesola balance to the nearest 0.2 g every day prior to presentation to each individual. The same protocol was followed when weighing uneaten food (for details, see Kwieciński et al. 2006a, b). The mean mass of each prey type and its percentage contribution to overall diet are presented in Table 1.

**Table 1:** Food consumption of investigated white storks (*N* = 29). The table shows mean daily mass (±SD) of food items consumed per white stork individual and the percentage contribution to the overall diet based on mass

Taxa	Males *N* = 9		Females *N* = 20	
	Mass (g)	Proportion in diet (%)	Mass (g)	Proportion in diet (%)
Mammals	84.91 ± 33.29	34.77	70.52 ± 39.76	23.26
Birds	107.02 ± 61.48	43.83	183.27 ± 119.45	60.45
Amphibians	5.01 ± 9.83	2.05	3.61 ± 7.18	1.19
Fish	39.89 ± 19.41	16.34	42.86 ± 13.76	14.14
Insects	5.12 ± 5.22	2.10	2.60 ± 2.30	0.86
Earthworms	2.24 ± 4.15	0.92	0.34 ± 1.06	0.11

### Food preferences of white storks

In the experiment where all food types were offered together in a single arena, we measured selectivity for all of the food types by calculating an index for selectivity (Larrinaga 2010), using the formula:

$$ {X}_{ijk}=\frac{t_{ijk}0-{t}_{ijk}1}{\sum_{k=1}^n\left({t}_{ijk}0-{t}_{ijk}1\right)/ n} $$

where *X*_*ijk*_ is the preference value of the subject (individual bird) *i* for prey type *k* in trial replicate *j*, *t*_*ijk*_1 is the weight of uneaten *k*-prey at the end of the trial, *t*_*ijk*_0 is the weight of available *k*-prey at the beginning of the trial and *n* is the number of food types included in the experiment. The magnitude of *X* indicates the degree of preference. Thus, values of *X* >1 indicate a relative preference for prey types *k*, while values of *X* <1 indicate relative avoidance. For every individual (males, *N* = 9; females, *N* = 20), 10 trials were performed (290 in total). Trials were conducted over 10 consecutive days (one trial during 1 day per individual bird).

### Sequence of food choice

We also examined the sequence in which particular food items were chosen by the examined birds. The food choices were classified into six categories (1—particular prey type as a first choice, 2—as a second choice and so on). During the experiment, males made 90 first choices (10 days × 9 males) and females made 200 choices (10 days × 20 females). Some individuals stopped feeding after the first choice; thus, the number of second and subsequent choices in the following categories may not add up to 90 in the case of males or 200 in the case of females.

### Foraging duration

During the observations, the time and duration of each individual’s feeding was recorded. Recording started when the food was presented and finished 4 h later; foraging activity was expressed in minutes of foraging duration on each food type.

### Statistical analysis

Where necessary, we used data transformation (logarithm) to obtain a normal distribution of residuals in dependent variables. We used mean values of 10 replicates for each food type.

We used a general linear model (GLM) with multiple dependent variables to test the difference between males and females in preference for prey type and in foraging time. We calculated the GLM using the Gaussian distribution; the dependent variables were food types: mammal, bird, amphibian, fish, earthworm and insect.

Sex differences in the sequence of food choices by white storks were tested using canonical correspondence analysis. Food choice was scored for each prey category and ranged as follows: first choice, a score of 1; second choice, 0.5; third choice, 0.25; fourth choice, 0.125; fifth choice, 0.0625; and sixth choice, 0.0312.

Calculations were conducted using the package Canoco 4.5 for Windows and SPSS 17.0. All basic statistical analyses followed the recommendations by Zarr (1999).

## Results

### Sex-related food type preference

Males preferred to eat birds (2.415 ± 0.444) and mammals (2.248 ± 0.363). The third preferred food item was insects (0.296 ± 0.169). The remaining food types represented marginally preferences on the part of males (amphibians 0.139 ± 0.092, fish 0.118 ± 0.095 and earthworms 0.077 ± 0.0460). The females preferred birds (3.397 ± 0.273) and mammals (1.467 ± 0.208). The amphibians were the third food choice of females (0.094 ± 0.041). As in the case of males, the remaining food types were slightly preferred by females (insects 0.072 ± 0.018, fish 0.025 ± 0.003 and earthworms 0.01 ± 0.007).

Differences in preferred food type between sexes were significant for the bird (*F* = 4.388, *P* = 0.046, Table 2). Females preferred birds (3.397 ± 0.273) more than males did (2.415 ± 0.444).

**Table 2:** The results of the GLM multiple dependent variables testing differences on preferences of each food type between males and females

Food types	*df*	MS	*F*	*P*
Mammals	1, 27	0.194	3.315	0.080
Birds	1, 27	0.257	4.388	0.046
Amphibians	1, 27	0.013	0.270	0.607
Fish	1, 27	0.800	0.459	0.504
Earthworms	1, 27	0.750	3.725	0.064
Insects	1, 27	0.016	0.040	0.843

### Food choice sequence

**Fig. 2:**
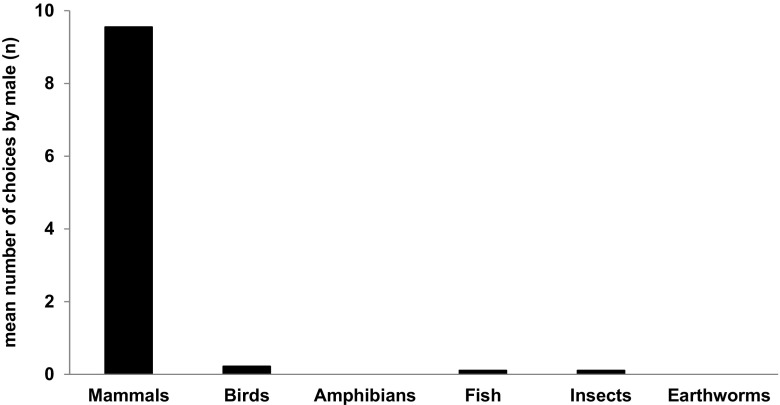
First choices of white stork males. *Bars* show the mean number of times when males chose a particular type of prey as first (*N* = 90; 10 days × 9 males)

Mammals were the prey type chosen first (prey category 1) the greatest number of times (86) by males (Fig. 2). As the second choice (prey category 2), birds were selected 40 times, fish 30, amphibians 10 and insects 6 times by white stork males. Additionally, mammals were the second choice of the tested individuals, and insects constituted prey category 2 only for 1 male white stork. Insects were the third prey category for 25 white stork males, birds for 24, fish for 8, amphibians for 4, earthworms for 2 and mammals for 1 of the tested individuals. As a fourth choice, 31 males selected insects and 10 earthworms.

**Fig. 3:**
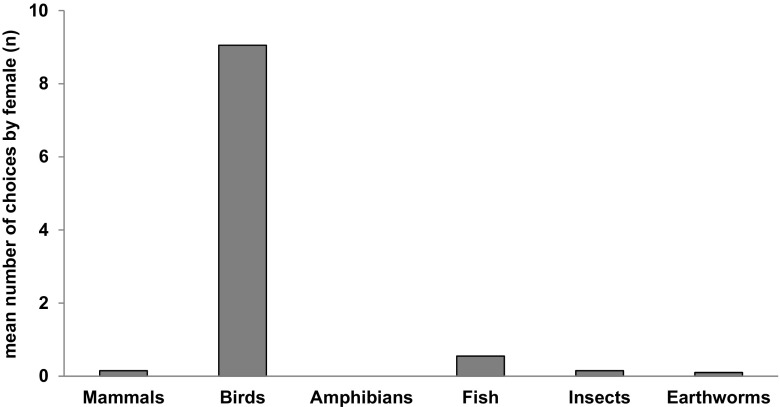
First choices of white stork females. *Bars* show the mean number of times when females chose a particular type of prey as first (*N* = 200; 10 days × 20 females)

Among females, birds were the prey type chosen first (prey category 1) the greatest number of times (181, Fig. 3). As the second choice (prey category 2), mammals were selected by females 77 times, fish 63, birds 16, amphibians 5, insects 2 and earthworms 1. Mammals constituted the third prey category for 64 white stork females, fish for 20, amphibians for 12 and insects for 5. As a fourth choice, 37 females selected insects, 7 mammals and 7 amphibians.

**Fig. 4:**
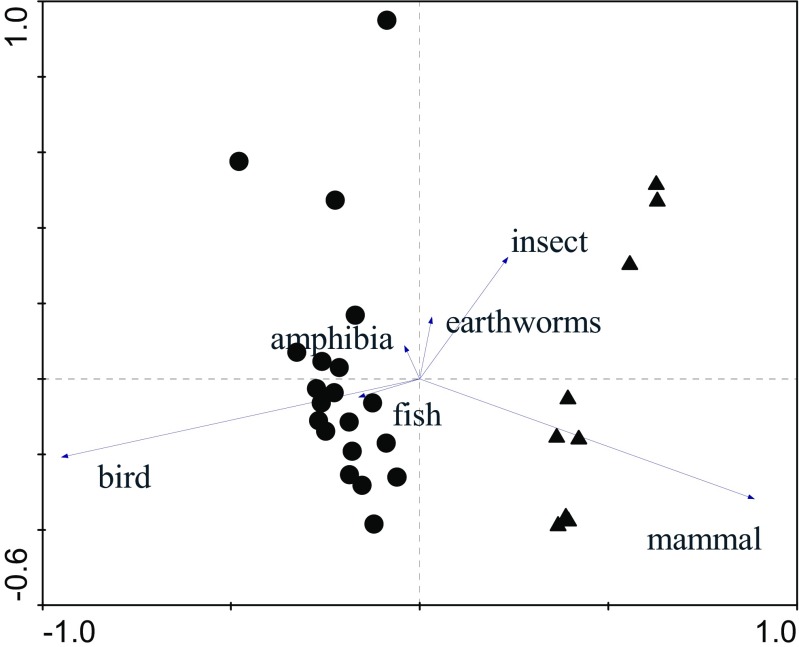
Correspondence analysis biplot of order scores achieved by particular prey types. The *arrows* denote a particular score of preference for prey type. Females are marked as *circles*, males as *triangles*. Increasing distance between points indicates greater dissimilarity of food preferences between individual birds. The model is significant *F* = 571.96, *P* = 0.002, eigenvalue of first axis = 0.963

Canonical correspondence analysis showed statistically significant differences between males and females for mammals (*F* = 2.98, *P* = 0.04), birds (*F* = 17.62, *P* = 0.002) and amphibians (*F* = 5.76, *P* = 0.03). A significantly higher number of males, compared to females, chose mammals as their first prey. A higher number of females, compared to males, selected birds (Fig. 4).

### Foraging duration of the white stork

Males spent significantly less time on foraging than females (16:16 ± 0:14 vs 87:12 ± 7:39 min, respectively; *F*_1, 27_ = 115.29; *P* < 0.001).

## Discussion

We have shown that small body size differences between the sexes in white storks were indicative of potentially considerable differences in foraging behaviour and some food preferences between sexes in our study.

The observed differences in diet between sexes may reflect their different behaviours and needs during their lifetime. The experiment was carried out during the breeding season (May, June). At this time, the behaviour and physiology of white stork females in the wild is linked with production and incubation of relatively large eggs, brood care that results in spending much more time in the nest than males, whereas the latter mainly defend territories and deliver food to their nests (Collopy 1984; Hawkins 1986; Sasvári and Hegyi 2001; Deeming 2002b; Bocheński and Jerzak 2006; Kosicki 2010, 2012; Tobółka et al. 2015; Żołnierewicz et al. 2016). Thus, it is possible that the preferences of female white storks for avian prey might be an indication for fast supplementation of calcium and other nutrients which are more readily available from avian skeletons than from mammalian (Bilby and Widdowson 1971; Graveland and van Gijzen 1994; Poulini and Brigham 2001; Reynolds et al. 2004). This may be an important strategy during spring (return from wintering ground and egg laying and incubation) which requires substantial amounts of microelements and energy (Walsberg 1983; Neger et al. 2001; Reid et al. 2002; Tinbergen and Williams 2002; Durant et al. 2004; Neger 2006; Kitowski 2007; Djerdali et al. 2008, 2016; Wuczyński 2012; Chenchouni et al. 2015; Chenchouni 2016). Despite studied females did not undertake attempts to reproduce or lay eggs before or after the completion of the study, the general physiological processes in wild and captive birds, including white storks, are similar (Hall et al. 1987; Herborn et al. 2010).

The divergent foraging patterns of white stork males and females were also observable in differences in the duration of foraging, with males spending less time on this activity than females. In our studies, the differences in foraging time between males and females amounted to 1:4. Other studies had found that males have a higher capture rate and deliver most food during the incubation and rearing of chicks (Collopy 1984; Hawkins 1986; Sasvári and Hegyi 2001; Matysioková and Reme 2010; Matysioková et al. 2011). Thus, it is advantageous for them to hunt and digest quickly. Females can be more selective in prey choice during foraging, and thus, their foraging trips may last longer. Moreover, both sexes differ significantly in intestine length, with females having longer intestines than males that result in the production of significantly fewer pellets than males (Kwieciński, unpublished data; Rosin and Kwieciński 2011).

Recent observations have shown no significant differences in foraging time between adult and juvenile storks during their migration to wintering grounds (Rotics et al. 2016).

Moreover, we cannot exclude the possibility that the results obtained in captivity may not fully reflect the behaviour of birds (Cieślak and Kwieciński 2009). Unnatural conditions such as limited flight space, sustainable provision of food and permanent contact with humans can modify the behaviour of birds.

The general dietary composition of the studied white storks was similar to that determined in field conditions: mammals were taken in great numbers, followed by birds and fish (Pinowska and Pinowski 1989; Pinowski et al. 1991; Antczak et al. 2002; Kosicki 2010, 2012; Chenchouni et al. 2015; Tobółka et al. 2015; Chenchouni 2016). Amphibians, traditionally considered common prey for the white stork, contribute to its diet to a variable degree depending mainly on weather conditions (Schierer 1967; Pikulik et al. 2001; Antczak et al. 2002; Profus 2006; Kosicki et al. 2006) but in experimental conditions were eaten rather rarely. Fish were preferred to amphibians; in the wild, fish contribute to the white stork’s diet mainly in fishpond areas (Profus 2006). Fish constitute a valuable source of energy and protein, thus are used successfully as a standard diet for captive storks (Dierenfeld et al. 2002; Kwieciński et al. 2006b).

Among invertebrates, insects and earthworms are a very important component of the diet of wild white storks (Hornberger 1967; Alonso et al. 1991; Pinowska et al. 1991; Profus 2006; Kosicki et al. 2006; Orłowski et al. 2016). However, our experiment showed that invertebrates are neglected by storks when vertebrate prey is available. This may be due to the fact that arthropods provide less energy and proteins per 1 g of fresh mass than mammals and birds (Górecki 1967; Dolnik et al. 1982; Barton and Houston 1993; Dierenfeld et al. 2002; Rosin and Kwieciński 2011; Chenchouni et al. 2015). However, the common occurrence of arthropods in the diet of white storks noted in field studies indicates that this prey type may be simply the most abundant in the wild and therefore the easiest to find.

To the best of our best knowledge, the current study is the first experimental test of food preferences in the white stork. Our study suggests that the calorific and protein content of consumed prey, apart from its availability, plays an important role in food selection in white storks. The results also intimate that there are sex differences in food preferences. These differences may have caused differences in general behaviour, anatomy (intestine length) and physiology (calcium management, pellet production rate). Our results add not only to the understanding of sex differences in food preferences but also to that of conservation of white storks. Studies on nutritional needs and food preferences play a crucial role in the conservation of threatened species (Fasta-Bianchet and Apollonio 2003; Olsson 2007). The white stork is an endangered, iconic species in Europe. Therefore, an important practice in white stork protection is to conserve and possibly supply habitats with valuable food such as small mammals and birds (Schulz 1998; Tryjanowski and Kuźniak 2002; Denac 2006; Djerdali et al. 2008; Hušek et al. 2013; Kwieciński et al. 2016).

## Electronic supplementary material

ESM 1 (PDF 726 kb)
