# Supplementary material for: Sexual differences in food preferences in the white stork: an experimental study
Source: Naturwissenschaften. 2017 Apr 7;104(5):39. doi: 10.1007/s00114-017-1457-5 (PMC5384956; doi:10.1007/s00114-017-1457-5)
Supplement: Supplementary file 1 — (PDF 726 kb) [file 114_2017_1457_MOESM1_ESM.pdf]

Data raw 1. Food preferences of White Storks – source data.

sex.	id	T1MAM	T2MAM	T3MAM	T4MAM	T5MAM	T6MAM	T7MAM	T8MAM	T9MAM	T10MAM
MALE	1,00	90,00	165,50	105,00	105,00	117,50	96,50	105,50	110,00	121,50	196,50
MALE	2,00	87,00	139,50	109,50	108,00	114,50	106,50	98,50	105,50	129,00	119,00
MALE	3,00	51,50	161,50	143,00	76,50	132,00	82,00	106,00	132,50	116,00	113,00
MALE	4,00	137,50	165,50	138,00	125,50	145,00	123,00	14,00	108,50	70,00	107,00
MALE	5,00	63,00	42,00	38,50	43,50	88,50	0,00	0,00	14,00	21,00	143,00
MALE	6,00	103,00	105,00	112,50	98,50	127,00	63,00	20,00	34,00	93,00	116,00
MALE	7,00	120,50	145,50	99,50	93,00	99,00	12,50	44,00	35,00	67,00	96,00
MALE	8,00	105,50	106,50	0,00	0,00	0,00	23,50	20,00	15,50	0,00	0,00
MALE	9,00	116,50	123,50	105,50	23,00	85,00	0,00	0,00	16,00	93,00	92,00
FEMALE	10,00	173,50	104,00	109,00	124,50	141,50	200,00	198,00	149,00	137,00	200,00
FEMALE	11,00	171,00	114,00	129,00	120,50	128,00	171,00	152,50	113,50	140,00	200,00
FEMALE	12,00	28,50	64,00	26,50	0,00	66,00	53,00	62,50	32,50	72,00	50,50
FEMALE	13,00	128,50	114,00	107,00	112,00	101,00	51,50	15,50	31,50	22,50	23,50
FEMALE	14,00	121,00	111,50	111,00	107,00	115,50	26,00	16,00	38,50	0,00	0,00
FEMALE	15,00	0,00	0,00	0,00	0,00	0,00	43,50	75,00	64,50	19,00	22,50
FEMALE	16,00	14,50	0,00	62,00	62,50	88,50	0,00	7,50	0,00	0,00	0,00
FEMALE	17,00	0,00	24,00	40,50	0,00	0,00	50,50	102,50	98,00	105,50	91,50
FEMALE	18,00	134,50	190,50	172,00	139,00	155,50	25,50	42,00	99,00	51,00	57,50
FEMALE	19,00	22,00	0,00	0,00	0,00	67,50	0,00	30,50	66,50	106,50	65,50
FEMALE	20,00	63,00	0,00	0,00	0,00	0,00	0,00	20,50	74,00	107,50	43,50
FEMALE	21,00	123,50	117,00	140,00	143,00	143,00	52,50	49,50	62,00	77,00	110,50
FEMALE	22,00	0,00	0,00	0,00	0,00	0,00	0,00	0,00	0,00	0,00	0,00
FEMALE	23,00	107,00	132,50	103,00	114,00	112,00	0,00	0,00	0,00	76,50	94,50
FEMALE	24,00	151,00	113,50	140,50	86,50	83,50	0,00	29,00	49,00	25,50	92,50
FEMALE	25,00	194,50	191,00	118,00	31,00	200,00	18,00	10,00	15,00	9,50	0,00
FEMALE	26,00	189,00	200,00	87,50	31,50	200,00	0,00	0,00	0,00	11,00	0,00

FEMALE	27,00	197,50	200,00	104,00	34,00	190,50	91,00	99,00	28,50	40,50	49,00
FEMALE	28,00	200,00	200,00	16,00	0,00	116,50	0,00	17,50	0,00	0,00	0,00
FEMALE	29,00	200,00	200,00	108,00	36,00	200,00	29,00	56,50	0,00	0,00	0,00

sex.	id	T1BIRDS	T2BIRDS	T3BIRDS	T4BIRDS	T5BIRDS	T6BIRDS	T7BIRDS	T8BIRDS	T9BIRDS	T10BIRDS
MALE	1,00	105,50	0,00	48,50	0,00	129,00	0,00	0,00	0,00	0,00	0,00
MALE	2,00	0,00	0,00	82,50	36,00	70,00	0,00	0,00	0,00	0,00	0,00
MALE	3,00	151,00	75,00	66,50	39,50	71,00	4,00	184,00	179,50	153,50	166,50
MALE	4,00	38,50	38,50	154,50	155,00	113,00	0,00	168,50	167,50	180,00	183,00
MALE	5,00	77,00	0,00	0,00	0,00	0,00	0,00	0,00	121,00	184,00	165,50
MALE	6,00	169,00	164,00	192,00	154,00	172,00	197,00	177,00	161,50	175,50	169,50
MALE	7,00	173,00	39,50	43,00	0,00	0,00	56,50	172,00	173,50	200,00	200,00
MALE	8,00	162,00	156,50	165,50	131,00	87,50	100,50	165,00	180,00	200,00	200,00
MALE	9,00	77,50	87,50	90,00	189,00	192,00	188,00	200,00	200,00	200,00	200,00
FEMALE	10,00	169,50	135,50	180,00	155,00	192,50	0,00	0,00	0,00	0,00	0,00
FEMALE	11,00	166,50	129,50	160,00	122,50	200,00	0,00	0,00	0,00	0,00	0,00
FEMALE	12,00	145,00	185,50	168,50	173,50	164,00	173,00	146,50	145,00	159,50	200,00
FEMALE	13,00	0,00	0,00	0,00	0,00	0,00	162,50	174,00	172,00	172,50	200,00
FEMALE	14,00	36,50	0,00	103,50	0,00	35,50	129,50	165,50	200,00	186,00	200,00
FEMALE	15,00	0,50	45,50	123,50	136,50	48,50	144,50	163,50	171,00	182,50	168,00
FEMALE	16,00	0,00	0,00	48,50	0,00	0,00	29,50	90,00	142,00	176,00	184,50
FEMALE	17,00	0,00	83,00	170,50	0,00	48,00	5,00	0,00	63,50	73,50	0,00
FEMALE	18,00	154,00	160,50	200,00	46,50	92,00	179,50	181,00	200,00	191,00	198,50
FEMALE	19,00	181,50	200,00	196,00	152,00	89,00	193,00	200,00	184,50	200,00	200,00
FEMALE	20,00	176,50	200,00	176,50	113,50	163,00	189,50	200,00	194,50	200,00	200,00
FEMALE	21,00	131,00	191,50	188,00	89,00	153,50	190,00	200,00	193,00	181,00	200,00
FEMALE	22,00	177,50	170,50	175,50	169,00	157,50	146,50	200,00	200,00	200,00	200,00
FEMALE	23,00	187,50	53,50	0,00	26,50	89,50	0,00	168,50	200,00	200,00	200,00
FEMALE	24,00	0,00	0,00	0,00	0,00	0,00	0,00	83,50	98,00	106,50	166,00

FEMALE	25,00	189,50	200,00	200,00	200,00	196,00	200,00	200,00	200,00	200,00	200,00
FEMALE	26,00	196,50	200,00	200,00	200,00	54,00	200,00	200,00	200,00	200,00	200,00
FEMALE	27,00	200,00	200,00	200,00	200,00	200,00	200,00	200,00	200,00	200,00	200,00
FEMALE	28,00	200,00	200,00	200,00	200,00	200,00	200,00	200,00	200,00	200,00	200,00
FEMALE	29,00	200,00	200,00	200,00	200,00	200,00	200,00	200,00	200,00	200,00	200,00

sex.	id	T1AMPHIBIAN	T2AMPHIBIAN	T3AMPHIBIAN	T4AMPHIBIAN	T5AMPHIBIAN	T6AMPHIBIAN	T7AMPHIBIAN	T8AMPHIBIAN	T9AMPHIBIAN	T10AMPHIBIAN
MALE	1,00	0,00	0,00	0,00	0,00	0,00	62,50	48,50	64,00	65,50	56,00
MALE	2,00	0,00	0,00	0,00	0,00	0,00	0,00	0,50	30,50	37,00	11,00
MALE	3,00	0,00	0,00	0,00	0,00	0,00	0,00	22,50	0,00	33,00	19,50
MALE	4,00	0,00	0,00	0,00	0,00	0,00	0,00	0,00	0,00	0,00	0,00
MALE	5,00	0,00	0,00	0,00	0,00	0,00	0,00	0,00	0,00	0,00	0,00
MALE	6,00	0,00	0,00	0,00	0,00	0,00	0,00	0,00	0,00	0,00	0,00
MALE	7,00	0,00	0,00	0,00	0,00	0,00	0,00	0,00	0,00	0,00	0,00
MALE	8,00	0,00	0,00	0,00	0,00	0,00	0,00	0,00	0,00	0,00	0,00
MALE	9,00	0,00	0,00	0,00	0,00	0,00	0,00	0,00	0,00	0,00	0,00
FEMALE	10,00	0,00	0,00	0,00	0,00	0,00	60,50	52,50	73,00	48,50	49,50
FEMALE	11,00	0,00	0,00	0,00	0,00	0,00	34,00	26,00	18,50	46,00	0,00
FEMALE	12,00	0,00	0,00	0,00	0,00	0,00	0,00	35,00	33,50	31,50	40,50
FEMALE	13,00	0,00	0,00	0,00	0,00	0,00	0,00	8,00	32,00	0,00	0,00
FEMALE	14,00	0,00	0,00	0,00	0,00	0,00	10,00	6,50	17,50	18,50	11,50
FEMALE	15,00	0,00	0,00	0,00	0,00	0,00	0,00	0,00	0,00	0,00	0,00
FEMALE	16,00	0,00	0,00	0,00	0,00	0,00	0,00	0,00	0,00	0,00	0,00
FEMALE	17,00	0,00	0,00	0,00	0,00	0,00	0,00	0,00	0,00	0,00	0,00
FEMALE	18,00	0,00	0,00	0,00	0,00	0,00	9,50	10,00	6,50	6,50	14,50
FEMALE	19,00	0,00	0,00	0,00	0,00	0,00	0,00	0,00	0,00	5,00	0,00
FEMALE	20,00	0,00	0,00	0,00	0,00	0,00	0,00	7,00	9,50	0,00	0,00
FEMALE	21,00	0,00	0,00	0,00	0,00	0,00	0,00	0,00	0,00	0,00	0,00
FEMALE	22,00	0,00	0,00	0,00	0,00	0,00	0,00	0,00	0,00	0,00	0,00

FEMALE	23,00	0,00	0,00	0,00	0,00	0,00	0,00	0,00	0,00	0,00	0,00
FEMALE	24,00	0,00	0,00	0,00	0,00	0,00	0,00	0,00	0,00	0,00	0,00
FEMALE	25,00	0,00	0,00	0,00	0,00	0,00	0,00	0,00	0,00	0,00	0,00
FEMALE	26,00	0,00	0,00	0,00	0,00	0,00	0,00	0,00	0,00	0,00	0,00
FEMALE	27,00	0,00	0,00	0,00	0,00	0,00	0,00	0,00	0,00	0,00	0,00
FEMALE	28,00	0,00	0,00	0,00	0,00	0,00	0,00	0,00	0,00	0,00	0,00
FEMALE	29,00	0,00	0,00	0,00	0,00	0,00	0,00	0,00	0,00	0,00	0,00

sex.	id	T1FISH	T2FISH	T3FISH	T4FISH	T5FISH	T6FISH	T7FISH	T8FISH	T9FISH	T10FISH
MALE	1,00	53,00	128,50	75,50	58,50	81,50	0,00	0,00	0,00	0,00	0,00
MALE	2,00	0,00	6,50	8,50	0,00	15,00	0,00	0,00	0,00	0,00	0,00
MALE	3,00	77,00	97,00	115,50	110,00	84,50	0,00	0,00	0,00	0,00	0,00
MALE	4,00	73,50	95,50	112,50	132,50	95,50	0,00	0,00	0,00	0,00	0,00
MALE	5,00	79,00	0,00	5,50	16,00	0,00	0,00	0,00	0,00	0,00	0,00
MALE	6,00	86,00	133,50	103,00	104,50	109,00	0,00	0,00	0,00	0,00	0,00
MALE	7,00	105,00	107,50	73,00	107,50	108,00	0,00	0,00	0,00	0,00	0,00
MALE	8,00	96,50	96,00	108,00	105,00	107,00	0,00	0,00	0,00	0,00	0,00
MALE	9,00	102,00	102,50	106,00	110,00	100,00	0,00	0,00	0,00	0,00	0,00
FEMALE	10,00	88,00	0,00	74,00	69,60	72,50	0,00	0,00	0,00	0,00	0,00
FEMALE	11,00	76,50	59,00	7,00	58,00	52,00	0,00	0,00	0,00	0,00	0,00
FEMALE	12,00	0,00	39,50	111,00	40,50	69,50	0,00	0,00	0,00	0,00	0,00
FEMALE	13,00	100,50	90,50	82,00	113,00	112,50	0,00	0,00	0,00	0,00	0,00
FEMALE	14,00	100,00	99,50	143,50	101,00	100,50	0,00	0,00	0,00	0,00	0,00
FEMALE	15,00	45,00	0,00	0,00	73,50	83,00	0,00	0,00	0,00	0,00	0,00
FEMALE	16,00	95,50	41,00	91,00	87,50	0,00	0,00	0,00	0,00	0,00	0,00
FEMALE	17,00	102,50	101,50	0,00	103,50	102,00	0,00	0,00	0,00	0,00	0,00
FEMALE	18,00	128,50	53,00	20,00	105,00	104,00	0,00	0,00	0,00	0,00	0,00
FEMALE	19,00	111,00	104,00	106,50	100,50	103,50	0,00	0,00	0,00	0,00	0,00
FEMALE	20,00	117,00	104,50	103,00	101,50	104,50	0,00	0,00	0,00	0,00	0,00

FEMALE	21,00	108,50	107,50	90,50	108,00	74,50	0,00	0,00	0,00	0,00	0,00
FEMALE	22,00	105,50	106,00	111,00	111,50	114,00	0,00	0,00	0,00	0,00	0,00
FEMALE	23,00	100,00	109,50	105,50	112,50	106,00	0,00	0,00	0,00	0,00	0,00
FEMALE	24,00	104,50	103,00	102,50	108,50	105,00	0,00	0,00	0,00	0,00	0,00
FEMALE	25,00	146,50	151,00	118,00	200,00	56,00	0,00	0,00	0,00	0,00	0,00
FEMALE	26,00	155,00	39,50	23,50	0,00	0,00	0,00	0,00	0,00	0,00	0,00
FEMALE	27,00	142,50	153,50	62,00	91,00	154,00	0,00	0,00	0,00	0,00	0,00
FEMALE	28,00	141,00	182,50	22,00	20,00	0,00	0,00	0,00	0,00	0,00	0,00
FEMALE	29,00	0,00	161,50	62,00	55,50	84,00	0,00	0,00	0,00	0,00	0,00

sex.	id	T1INSECT	T2INSECT	T3INSECT	T4INSECT	T5INSECT	T6INSECT	T7INSECT	T8INSECT	T9INSECT	T10INSECT
MALE	1,00	22,00	18,00	18,50	21,00	21,00	2,50	20,50	11,00	19,00	5,50
MALE	2,00	12,50	18,50	19,50	16,00	18,00	7,00	4,50	7,00	10,50	1,50
MALE	3,00	2,00	4,00	0,50	2,00	3,50	0,00	3,00	2,50	0,50	1,50
MALE	4,00	1,50	3,50	2,50	2,50	3,50	0,00	4,00	3,00	2,00	2,00
MALE	5,00	12,50	16,00	8,00	4,50	3,00	2,50	1,00	3,50	2,00	1,50
MALE	6,00	7,50	3,50	3,50	12,00	8,50	1,50	1,00	1,00	1,50	3,50
MALE	7,00	0,00	1,50	2,00	1,00	1,50	0,50	0,00	0,00	0,50	0,00
MALE	8,00	2,00	1,50	0,00	1,00	1,50	0,00	0,50	0,00	0,00	0,50
MALE	9,00	3,00	5,00	4,00	8,00	4,50	1,50	1,00	2,00	1,50	1,00
FEMALE	10,00	1,50	0,50	7,00	8,00	4,50	1,50	1,00	0,50	1,50	0,50
FEMALE	11,00	4,50	1,00	2,50	3,00	2,00	1,00	1,00	1,00	1,50	1,50
FEMALE	12,00	0,00	1,50	3,00	2,00	2,00	2,00	1,00	0,00	0,00	0,50
FEMALE	13,00	1,00	2,00	4,00	2,50	2,00	1,50	2,00	2,50	1,00	1,00
FEMALE	14,00	8,00	2,00	2,00	5,50	4,50	1,50	1,00	3,50	2,00	1,50
FEMALE	15,00	5,50	6,50	2,00	2,50	1,00	0,50	0,00	1,00	1,50	1,50
FEMALE	16,00	4,00	1,00	1,00	1,00	0,50	0,00	2,00	1,50	1,00	3,00
FEMALE	17,00	16,00	14,00	10,50	24,00	7,00	2,50	0,50	2,00	1,50	0,00
FEMALE	18,00	2,50	3,00	1,00	2,00	1,50	0,50	0,50	1,00	1,50	0,00

FEMALE	19,00	18,50	6,00	8,00	6,50	6,00	1,50	0,50	2,00	0,50	0,50
FEMALE	20,00	16,00	12,50	4,50	11,00	12,00	0,00	3,50	2,50	2,00	1,50
FEMALE	21,00	1,00	1,00	6,00	5,00	0,50	0,00	0,00	0,50	0,00	0,00
FEMALE	22,00	4,50	7,00	5,00	16,50	23,00	0,00	0,00	2,50	0,00	4,00
FEMALE	23,00	3,50	1,50	2,50	8,00	5,50	0,00	1,50	0,50	1,00	0,50
FEMALE	24,00	3,00	23,00	2,00	16,00	2,50	0,00	1,50	0,50	0,00	0,00
FEMALE	25,00	7,50	0,00	0,00	0,00	0,00	0,00	0,00	0,00	0,00	0,00
FEMALE	26,00	0,00	0,00	0,00	0,00	0,00	0,00	0,00	0,00	0,00	0,00
FEMALE	27,00	10,00	0,00	0,00	0,00	0,00	0,00	0,00	0,00	0,00	0,00
FEMALE	28,00	0,00	0,00	0,00	0,00	0,00	0,00	0,00	0,00	0,00	0,00
FEMALE	29,00	0,00	0,00	0,00	0,00	0,00	0,00	0,00	0,00	0,00	0,00

sex.	id	T1EARTWORM	T2EARTWORM	T3EARTWORM	T4EARTWORM	T5EARTWORM	T6EARTWORM	T7EARTWORM	T8EARTWORM	T9EARTWORM	T10EARTWORM
MALE	1,00	0,00	0,00	0,00	0,00	0,00	25,50	30,50	21,00	19,00	12,50
MALE	2,00	0,00	0,00	0,00	0,00	0,00	9,50	27,00	15,00	17,00	11,50
MALE	3,00	0,00	0,00	0,00	0,00	0,00	3,50	0,00	0,00	0,00	0,00
MALE	4,00	0,00	0,00	0,00	0,00	0,00	6,00	0,00	3,50	0,00	0,00
MALE	5,00	0,00	0,00	0,00	0,00	0,00	0,00	0,00	0,00	0,00	0,00
MALE	6,00	0,00	0,00	0,00	0,00	0,00	0,00	0,00	0,00	0,00	0,00
MALE	7,00	0,00	0,00	0,00	0,00	0,00	0,00	0,00	0,00	0,00	0,00
MALE	8,00	0,00	0,00	0,00	0,00	0,00	0,00	0,00	0,00	0,00	0,00
MALE	9,00	0,00	0,00	0,00	0,00	0,00	0,00	0,00	0,00	0,00	0,00
FEMALE	10,00	0,00	0,00	0,00	0,00	0,00	5,50	9,50	6,50	1,00	4,00
FEMALE	11,00	0,00	0,00	0,00	0,00	0,00	16,50	5,50	9,00	1,00	8,50
FEMALE	12,00	0,00	0,00	0,00	0,00	0,00	0,00	0,00	0,00	0,00	0,00
FEMALE	13,00	0,00	0,00	0,00	0,00	0,00	0,00	0,00	0,00	0,00	0,00
FEMALE	14,00	0,00	0,00	0,00	0,00	0,00	0,00	0,00	0,00	0,00	0,00
FEMALE	15,00	0,00	0,00	0,00	0,00	0,00	0,00	0,00	0,00	0,00	0,00
FEMALE	16,00	0,00	0,00	0,00	0,00	0,00	0,00	0,00	0,00	0,00	0,00

FEMALE	17,00	0,00	0,00	0,00	0,00	0,00	0,00	0,00	0,00	0,00	0,00
FEMALE	18,00	0,00	0,00	0,00	0,00	0,00	0,00	0,00	0,00	0,00	0,00
FEMALE	19,00	0,00	0,00	0,00	0,00	0,00	0,00	0,00	0,00	0,00	0,00
FEMALE	20,00	0,00	0,00	0,00	0,00	0,00	0,00	0,00	0,00	0,00	0,00
FEMALE	21,00	0,00	0,00	0,00	0,00	0,00	0,00	0,00	0,00	0,00	0,00
FEMALE	22,00	0,00	0,00	0,00	0,00	0,00	0,00	0,00	0,00	0,00	0,00
FEMALE	23,00	0,00	0,00	0,00	0,00	0,00	0,00	0,00	0,00	0,00	0,00
FEMALE	24,00	0,00	0,00	0,00	0,00	0,00	0,00	0,00	0,00	0,00	0,00
FEMALE	25,00	0,00	0,00	0,00	0,00	0,00	0,00	0,00	0,00	0,00	0,00
FEMALE	26,00	0,00	0,00	0,00	0,00	0,00	0,00	0,00	0,00	0,00	0,00
FEMALE	27,00	0,00	0,00	0,00	0,00	0,00	0,00	0,00	0,00	0,00	0,00
FEMALE	28,00	0,00	0,00	0,00	0,00	0,00	0,00	0,00	0,00	0,00	0,00
FEMALE	29,00	0,00	0,00	0,00	0,00	0,00	0,00	0,00	0,00	0,00	0,00

Data raw 2. The sequence of food choice – source.

ID White Storks	SEX	TRIAL	MAMMAL	BIRDS	AMPHIBIANS	FISH	INSECTS	EARTHWORMS		ID SEQUENCE OF FOOD CHOICE	RANK	
1	MALE	1	1	0,5	0	0,25	0,125	0		1	1	
1	MALE	2	1	0	0	0,5	0,25	0		2	0,5	
1	MALE	3	1	0,25	0	0,5	0,125	0		3	0,25	
1	MALE	4	1	0	0	0,25	0,5	0		4	0,125	
1	MALE	5	1	0,25	0	0,5	0,125	0		5	0,0625	
1	MALE	6	1	0	0,5	0	0,125	0,25		6	0,03125	
1	MALE	7	1	0	0,5	0	0,25	0,125				
1	MALE	8	1	0	0,5	0	0,25	0,125				
1	MALE	9	1	0	0,5	0	0,25	0,125				
1	MALE	10	1	0	0,5	0	0,25	0,125				
2	MALE	1	1	0	0	0	0,5	0				
2	MALE	2	1	0	0	0,5	0,25	0,125				
2	MALE	3	1	0,5	0	0,125	0,25	0				
2	MALE	4	1	0,5	0	0	0,25	0,125				
2	MALE	5	1	0,5	0	0,125	0,25	0				
2	MALE	6	1	0	0	0	0,5	0				
2	MALE	7	1	0	0,5	0	0,25	0,125				
2	MALE	8	1	0	0,5	0	0,125	0,25				
2	MALE	9	1	0	0,5	0	0,25	0,125				
2	MALE	10	1	0	0,5	0	0,25	0,125				
3	MALE	1	0,25	0,5	0	1	0,125	0				
3	MALE	2	1	0,25	0	0,5	0,125	0				
3	MALE	3	1	0,25	0	0,5	0,125	0				
3	MALE	4	1	0,25	0	0,5	0,125	0				

3	MALE	5	1	0,25	0	0,5	0,125	0				
3	MALE	6	1	0,5	0	0	0	0,25				
3	MALE	7	1	0,5	0,25	0	0,125	0				
3	MALE	8	1	0,5	0	0	0,25	0				
3	MALE	9	1	0,5	0,25	0	0	0				
3	MALE	10	1	0,5	0,25	0	0,125	0				
4	MALE	1	1	0,25	0	0,5	0,125	0				
4	MALE	2	1	0,25	0	0,5	0,125	0				
4	MALE	3	1	0,25	0	0,5	0,125	0				
4	MALE	4	1	0,5	0	0,25	0,125	0				
4	MALE	5	1	0,5	0	0,25	0,125	0				
4	MALE	6	1	0	0	0	0	0,5				
4	MALE	7	1	0,5	0	0	0,25	0				
4	MALE	8	1	0,5	0	0	0,25	0,125				
4	MALE	9	1	0,5	0	0	0,25	0				
4	MALE	10	0,5	1	0	0	0	0				
5	MALE	1	1	0,5	0	0,25	0,125	0				
5	MALE	2	1	0	0	0	0,5	0				
5	MALE	3	1	0	0	0,5	0,25	0				
5	MALE	4	1	0	0	0,5	0,25	0				
5	MALE	5	1	0	0	0	0,5	0				
5	MALE	6	0,5	0	0	0	1	0				
5	MALE	7	1	0	0	0	0	0				
5	MALE	8	1	0,5	0	0	0,25	0				
5	MALE	9	1	0,5	0	0	0,25	0				
5	MALE	10	1	0,5	0	0	0	0				
6	MALE	1	1	0,25	0	0,5	0	0				
6	MALE	2	1	0,25	0	0,5	0,125	0				
6	MALE	3	1	0,25	0	0,5	0,125	0				

6	MALE	4	1	0	0	0,5	0,25	0				
6	MALE	5	1	0	0	0,5	0	0				
6	MALE	6	0,5	1	0	0	0	0				
6	MALE	7	1	0,5	0	0	0	0				
6	MALE	8	1	0,5	0	0	0	0				
6	MALE	9	1	0,5	0	0	0	0				
6	MALE	10	1	0,5	0	0	0	0				
7	MALE	1	1	0,5	0	0,25	0,125	0				
7	MALE	2	1	0,5	0	0,25	0	0				
7	MALE	3	1	0,25	0	0,5	0	0				
7	MALE	4	1	0,125	0	0,5	0,25	0				
7	MALE	5	1	0,125	0	0,5	0,25	0				
7	MALE	6	1	0,5	0	0	0	0				
7	MALE	7	1	0,5	0	0	0	0				
7	MALE	8	1	0,5	0	0	0	0				
7	MALE	9	1	0,5	0	0	0	0				
7	MALE	10	1	0,5	0	0	0	0				
8	MALE	1	1	0,25	0	0,5	0,125	0				
8	MALE	2	1	0,25	0	0,5	0,125	0				
8	MALE	3	1	0,25	0	0,5	0,125	0				
8	MALE	4	1	0,5	0	0,25	0,125	0				
8	MALE	5	1	0,25	0	0,5	0,125	0				
8	MALE	6	1	0,25	0	0	0,5	0				
8	MALE	7	1	0,5	0	0	0	0				
8	MALE	8	1	0,5	0	0	0,25	0				
8	MALE	9	1	0,5	0	0	0,25	0				
8	MALE	10	1	0,5	0	0	0	0				
9	MALE	1	1	0,25	0	0,5	0,125	0				
9	MALE	2	1	0,25	0	0,5	0,125	0				

9	MALE	3	1	0,25	0	0,5	0,125	0				
9	MALE	4	1	0,25	0	0,5	0,125	0				
9	MALE	5	1	0,25	0	0,5	0,125	0				
9	MALE	6	1	0,5	0	0	0	0				
9	MALE	7	1	0,5	0	0	0	0				
9	MALE	8	1	0,5	0	0	0	0				
9	MALE	9	1	0,5	0	0	0	0				
9	MALE	10	1	0,5	0	0	0	0				
10	FEMALE	1	0,25	1	0	0,5	0,125	0				
10	FEMALE	2	0,25	1	0	0,5	0,125	0				
10	FEMALE	3	0,25	1	0	0,5	0,125	0				
10	FEMALE	4	0,25	1	0	0,5	0,125	0				
10	FEMALE	5	0,25	1	0	0,5	0,125	0				
10	FEMALE	6	0,125	1	0,25	0	0,5	0				
10	FEMALE	7	0,25	1	0,125	0	0	0,5				
10	FEMALE	8	0,25	1	0,125	0	0,5	0				
10	FEMALE	9	0,5	1	0,125	0	0,25	0				
10	FEMALE	10	0,125	1	0,5	0	0,25	0				
11	FEMALE	1	0,25	1	0	0,5	0,125	0				
11	FEMALE	2	0,25	1	0	0,5	0,125	0				
11	FEMALE	3	0,25	1	0	0,5	0,125	0				
11	FEMALE	4	0,25	1	0	0,5	0,125	0				
11	FEMALE	5	0,25	1	0	0,5	0,125	0				
11	FEMALE	6	0,25	0,5	0,125	0	1	0				
11	FEMALE	7	0,25	0,5	0,125	0	0	1				
11	FEMALE	8	0,25	0,5	0,125	0	1	0				
11	FEMALE	9	0,25	0,5	0	0	0	1				
11	FEMALE	10	0,25	0,5	0	0	1	0				
12	FEMALE	1	0,5	1	0	0,25	0,125	0				

12	FEMALE	2	0,5	1	0	0,25	0,125	0				
12	FEMALE	3	0,5	1	0	0,25	0,125	0				
12	FEMALE	4	0,5	1	0	0,25	0,125	0				
12	FEMALE	5	0,5	1	0	0,25	0,125	0				
12	FEMALE	6	0,5	1	0	0	0,125	0				
12	FEMALE	7	0,5	1	0,25	0	0,125	0				
12	FEMALE	8	0,5	1	0,25	0	0	0				
12	FEMALE	9	0,5	1	0,25	0	0	0				
12	FEMALE	10	0,5	1	0	0	0	0				
13	FEMALE	1	0,25	0,5	0	1	0	0				
13	FEMALE	2	0,25	0,5	0	1	0	0				
13	FEMALE	3	0,25	0,5	0	1	0	0				
13	FEMALE	4	0,25	0,5	0	1	0	0				
13	FEMALE	5	0,25	0,5	0	1	0	0				
13	FEMALE	6	0,25	1	0,5	0	0	0				
13	FEMALE	7	0,25	1	0,5	0	0	0				
13	FEMALE	8	0,25	1	0,5	0	0	0				
13	FEMALE	9	0,25	1	0	0	0,5	0				
13	FEMALE	10	0,5	1	0	0	0	0				
14	FEMALE	1	0,25	0,5	0	1	0,125	0				
14	FEMALE	2	0,25	0,5	0	1	0,125	0				
14	FEMALE	3	0,25	0,5	0	1	0,125	0				
14	FEMALE	4	0,25	0,5	0	1	0,125	0				
14	FEMALE	5	0,25	1	0	0,5	0	0				
14	FEMALE	6	0,5	1	0,25	0	0	0				
14	FEMALE	7	0,5	1	0,25	0	0	0				
14	FEMALE	8	0,5	1	0,25	0	0	0				
14	FEMALE	9	0,5	1	0	0	0	0				
14	FEMALE	10	0,5	1	0	0	0	0				

15	FEMALE	1	0	0	0	1	0,5	0				
15	FEMALE	2	0	1	0	0,5	0	0				
15	FEMALE	3	0	1	0	0,5	0	0				
15	FEMALE	4	0	1	0	0,5	0	0				
15	FEMALE	5	0	1	0	0,5	0	0				
15	FEMALE	6	0,5	1	0	0	0	0				
15	FEMALE	7	0,5	1	0	0	0	0				
15	FEMALE	8	0,5	1	0	0	0	0				
15	FEMALE	9	0,5	1	0	0	0	0				
15	FEMALE	10	0,5	1	0	0	0	0				
16	FEMALE	1	0,5	1	0	0,25	0,125	0				
16	FEMALE	2	0	1	0	0,5	0	0				
16	FEMALE	3	0,25	1	0	0,5	0,125	0				
16	FEMALE	4	0,25	1	0	0,5	0,125	0				
16	FEMALE	5	1	0,5	0	0,25	0	0				
16	FEMALE	6	0	1	0	0	0	0				
16	FEMALE	7	0	1	0	0	0	0				
16	FEMALE	8	0	1	0	0	0	0				
16	FEMALE	9	0	1	0	0	0	0				
16	FEMALE	10	0	1	0	0	0	0				
17	FEMALE	1	0,5	1	0	0,25	0,125	0				
17	FEMALE	2	0,25	0,5	0	1	0,125	0				
17	FEMALE	3	0,5	1	0	0,25	0,125	0				
17	FEMALE	4	0	1	0	0,5	0	0				
17	FEMALE	5	0	1	0	0	0	0				
17	FEMALE	6	0,5	1	0	0	0	0				
17	FEMALE	7	1	1	0	0	0	0				
17	FEMALE	8	0,5	1	0	0	0	0				
17	FEMALE	9	0,5	1	0	0	0	0				

17	FEMALE	10	1	0	0	0	0	0				
18	FEMALE	1	0,5	1	0	0,25	0,125	0				
18	FEMALE	2	0,25	1	0	0,5	0,125	0				
18	FEMALE	3	0,25	1	0	0,5	0,125	0				
18	FEMALE	4	0,25	1	0	0,5	0	0				
18	FEMALE	5	0,25	1	0	0,5	0	0				
18	FEMALE	6	0,5	1	0,25	0	0	0				
18	FEMALE	7	0,5	1	0,25	0	0	0				
18	FEMALE	8	0,5	1	0,25	0	0	0				
18	FEMALE	9	0,5	1	0,25	0	0	0				
18	FEMALE	10	0,25	1	0,5	0	0	0				
19	FEMALE	1	0,125	1	0	0,5	0,25	0				
19	FEMALE	2	0	1	0	0,5	0	0				
19	FEMALE	3	0	1	0	0,5	0	0				
19	FEMALE	4	0,125	1	0	0,5	0	0				
19	FEMALE	5	0,125	1	0	0,5	0	0				
19	FEMALE	6	0,125	1	0	0	0	0				
19	FEMALE	7	0,5	1	0	0	0	0				
19	FEMALE	8	0,5	1	0	0	0	0				
19	FEMALE	9	0,5	1	0,125	0	0	0				
19	FEMALE	10	0,5	1	0	0	0	0				
20	FEMALE	1	0,25	1	0	0,5	0	0				
20	FEMALE	2	0	1	0	0,5	0,25	0				
20	FEMALE	3	0	1	0	0,5	0	0				
20	FEMALE	4	0	1	0	0,5	0	0				
20	FEMALE	5	0	1	0	0,5	0	0				
20	FEMALE	6	0	1	0	0	0	0				
20	FEMALE	7	0,5	1	0,25	0	0	0				
20	FEMALE	8	0,5	1	0	0	0	0				

20	FEMALE	9	0,5	1	0	0	0	0				
20	FEMALE	10	0,5	1	0	0	0	0				
21	FEMALE	1	0,25	1	0	0,5	0,125	0				
21	FEMALE	2	0,25	1	0	0,5	0,125	0				
21	FEMALE	3	0,25	1	0	0,5	0	0				
21	FEMALE	4	0,25	1	0	0,5	0	0				
21	FEMALE	5	0,25	1	0	0,5	0	0				
21	FEMALE	6	0,5	1	0	0	0	0				
21	FEMALE	7	0,5	1	0	0	0	0				
21	FEMALE	8	0,5	1	0	0	0	0				
21	FEMALE	9	0,5	1	0	0	0	0				
21	FEMALE	10	0,5	1	0	0	0	0				
22	FEMALE	1	0	1	0	0,5	0,25	0				
22	FEMALE	2	0	1	0	0,5	0,25	0				
22	FEMALE	3	0	1	0	0,5	0,25	0				
22	FEMALE	4	0	1	0	0,5	0	0				
22	FEMALE	5	0	1	0	0,5	0	0				
22	FEMALE	6	0	1	0	0	0	0				
22	FEMALE	7	0	1	0	0	0	0				
22	FEMALE	8	0	1	0	0	0	0				
22	FEMALE	9	0	1	0	0	0	0				
22	FEMALE	10	0	1	0	0	0	0				
23	FEMALE	1	0,25	1	0	0,5	0	0				
23	FEMALE	2	0,25	1	0	0,5	0	0				
23	FEMALE	3	0,25	1	0	0,5	0,25	0				
23	FEMALE	4	0	1	0	0,5	0,25	0				
23	FEMALE	5	0	1	0	0	0	0				
23	FEMALE	6	0	1	0	0	0	0				
23	FEMALE	7	0,5	1	0	0	0	0				

23	FEMALE	8	0,5	1	0	0	0	0				
23	FEMALE	9	0,5	1	0	0	0	0				
23	FEMALE	10	0,5	1	0	0	0	0				
24	FEMALE	1	0,25	1	0	0,5	0,125	0				
24	FEMALE	2	0,25	1	0	0,5	0,125	0				
24	FEMALE	3	0,25	1	0	0,5	0,125	0				
24	FEMALE	4	0,25	1	0	0,5	0,125	0				
24	FEMALE	5	0,25	1	0	0,5	0	0				
24	FEMALE	6	0,5	1	0	0	0	0				
24	FEMALE	7	0,5	1	0	0	0	0				
24	FEMALE	8	0,5	1	0	0	0	0				
24	FEMALE	9	0,5	1	0	0	0	0				
24	FEMALE	10	0,5	1	0	0	0	0				
25	FEMALE	1	0,125	1	0	0,5	0,25	0				
25	FEMALE	2	0,25	1	0	0,5	0	0				
25	FEMALE	3	0,25	1	0	0,5	0	0				
25	FEMALE	4	0,25	1	0	0,5	0	0				
25	FEMALE	5	0,25	1	0	0,5	0	0				
25	FEMALE	6	0,25	1	0	0	0	0				
25	FEMALE	7	0,25	1	0	0	0	0				
25	FEMALE	8	0	1	0	0	0	0				
25	FEMALE	9	0	1	0	0	0	0				
25	FEMALE	10	0	1	0	0	0	0				
26	FEMALE	1	0,25	1	0	0,5	0	0				
26	FEMALE	2	0,5	1	0	0	0	0				
26	FEMALE	3	0,25	1	0	0,5	0	0				
26	FEMALE	4	0,5	1	0	0,25	0	0				
26	FEMALE	5	0,5	1	0	0	0	0				
26	FEMALE	6	0	1	0	0	0	0				

26	FEMALE	7	0	1	0	0	0	0				
26	FEMALE	8	0	1	0	0	0	0				
26	FEMALE	9	0,5	1	0	0	0	0				
26	FEMALE	10	0	1	0	0	0	0				
27	FEMALE	1	0,5	1	0	0,25	0,125	0				
27	FEMALE	2	0,5	1	0	0,25	0	0				
27	FEMALE	3	0,5	1	0	0,25	0	0				
27	FEMALE	4	0,25	1	0	0,5	0	0				
27	FEMALE	5	0,5	1	0	0,25	0	0				
27	FEMALE	6	0,5	1	0	0	0	0				
27	FEMALE	7	0,5	1	0	0	0	0				
27	FEMALE	8	0,5	1	0	0	0	0				
27	FEMALE	9	0,5	1	0	0	0	0				
27	FEMALE	10	0,5	1	0	0	0	0				
28	FEMALE	1	0,5	1	0	0,25	0	0				
28	FEMALE	2	0,5	1	0	0,25	0	0				
28	FEMALE	3	0,25	1	0	0,5	0	0				
28	FEMALE	4	0	1	0	0,5	0	0				
28	FEMALE	5	0,5	1	0	0	0	0				
28	FEMALE	6	0	1	0	0	0	0				
28	FEMALE	7	0,5	1	0	0	0	0				
28	FEMALE	8	0	1	0	0	0	0				
28	FEMALE	9	0	1	0	0	0	0				
28	FEMALE	10	0	1	0	0	0	0				
29	FEMALE	1	1	0,5	0	0	0	0				
29	FEMALE	2	0,5	1	0	0,25	0	0				
29	FEMALE	3	0,5	1	0	0,25	0	0				
29	FEMALE	4	0,25	1	0	0,5	0	0				
29	FEMALE	5	0,5	1	0	0,25	0	0				

29	FEMALE	6	0,5	1	0	0	0	0				
29	FEMALE	7	0,5	1	0	0	0	0				
29	FEMALE	8	0	1	0	0	0	0				
29	FEMALE	9	0	1	0	0	0	0				
29	FEMALE	10	0	1	0	0	0	0				
